# Efficacy and safety assessment of gelatin hemostatic matrix in a burr hole neurosurgical beagle model

**DOI:** 10.1038/s41598-025-28075-6

**Published:** 2025-12-23

**Authors:** Jun Ma, Shaolei Gan, Ruming Shu, Liangbin Zhou, Shangsi Chen

**Affiliations:** 1 Borayer Biotechnology Co. Ltd., Jiangxi, China; 2 Department of Biomedical Engineering, The Chinese University of Hong Kong, Shatin, N.T., Hong Kong, SAR China; 3 Department of Mechanical Engineering, The University of Hong Kong, Pokfulam Road, Hong Kong, SAR China

**Keywords:** Hemostatic matrix, Hemostasis, Gelatin, Cerebral hemorrhage, Beagle dogs, Diseases, Drug discovery, Medical research

## Abstract

Absorbable topical hemostatic agents are indispensable for managing excessive bleeding during neurosurgical and emergency procedures, significantly reducing the risk of serious complications. In the current study, we evaluated the hemostatic efficacy of two commercialized hemostatic agents, SURGIFLO (Ethicon, Johnson & Johnson, USA) and Borayerflo (Jiangxi Borayer Biotechnology Ltd, China), in a cerebral hemorrhage model using Beagle dogs. As a result, both SURGIFLO and Borayerflo achieved 100% hemostasis within 5 min of treatment, with no statistically significant differences observed between the two groups. Additionally, blood test results, including fibrinogen (FIB) level, thrombin time (TT), prothrombin time (PT), International Normalized Ratio (INR), and activated partial thromboplastin time (APTT), demonstrated that both SURGIFLO and Borayerflo matrices exhibited excellent blood compatibility and did not interfere with fibrinogen’s role in coagulation. Furthermore, toxicity test results indicated that no adverse reactions were observed post-operatively, confirming the safety of both SURGIFLO and Borayerflo. Consequently, the study validates the comparable safety and efficacy of the Borayerflo to SURGIFLO in terms of preclinical hemostatic efficacy and safety, making it a potential alternative product as a suitable absorbable topical hemostatic agent in the market.

## Introduction

Blood loss is an inevitable aspect of nearly all surgical procedures, making effective hemostasis a cornerstone for successful outcomes^1^. Precise bleeding control is essential not only for immediate surgical success but also for long-term patient recovery. The amount of blood loss during surgery depends on various factors, including the type of procedure, medications administered, and the methods used to achieve hemostasis. Failure to control bleeding can result in excessive blood loss, increasing the likelihood of blood transfusions, which may bring additional risks such as disease transmission and transfusion reactions. Furthermore, uncontrolled bleeding can complicate surgeries, prolong operative times, and lead to extended hospital stays—often up to 2 to 2.5 times longer for patients requiring transfusions^2^. Consequently, the importance of hemostasis is particularly pronounced in high-risk surgeries, such as neurosurgical procedures, where inadequate bleeding control is directly associated with higher rates of morbidity and mortality^3,4^. Therefore, hemostasis strategies to effectively manage surgical bleeding and minimize the need for transfusions are indispensable.

Rapid hemostasis at surgical sites is essential for minimizing blood loss, reducing perioperative morbidity, shortening operative times, and improving surgical outcomes^5^. Effective hemostasis is particularly critical in neurosurgical procedures, such as cranial and spinal surgeries. Substantial blood loss from the dura mater or surface vessels of the nervous tissue within the surgical cavity can compromise surgical outcomes^6^. In neurosurgery, conventional hemostasis methods, i.e., pressure, suturing, ligation, and monopolar or bipolar cauterization, may be ineffective or impractical^7–9^, especially when the bleeding source is difficult to identify or when there is an underlying or intraoperative coagulopathy. In these circumstances, topical hemostatic agents have emerged as valuable adjuncts. Previously, a variety of topical hemostatic agents have been developed, including gelatin foam, fibrin sealants, oxidized regenerated cellulose (ORC), and synthetic hydrogels and adhesives^7,10,11^.

Among them, despite the drawbacks including potential inflammatory reactions (e.g., granuloma formation in some formulations) and mechanical stiffness, which may limit use in delicate tissues, hemostatic products derived from animal-based gelatin (e.g., porcine and bovine) have gained widespread use in clinical practice due to their versatility across diverse surgical procedures, excellent biocompatibility, and minimal risk of adverse reactions^8,12–15^. They are easy to handle and adhere well to bleeding surfaces, making them ideal for neurosurgical and parenchymal applications. Flowable gelatin-based hemostatic agents, in particular, offer significant advantages over non-flowable counterparts, including the ability to conform to complex wound geometries, fill deep lesions, and allow for easy removal of excess material via irrigation^16,17^. These agents function effectively as topical hemostats by forming gels when exposed to a 0.9% saline solution^7,18^. Upon application to bleeding sites, flowable gelatin-based hemostatic agents facilitate the hemostatic cascade by promoting the conversion of fibrinogen to fibrin. Some products are also combined with thrombin to enhance their effectiveness, acting as physical adhesives to tissue while activating platelets and Factor XII, thus further supporting hemostasis^19–21^. Currently, several flowable gelatin-based hemostatic products, such as Floseal and SURGIFLO, have been commercialized and are widely used in clinical practice.

SURGIFLO hemostatic matrix is a flowable, gelatin-based surgical hemostatic product designed to effectively control bleeding during various surgical procedures. Manufactured by Ethicon, a subsidiary of Johnson & Johnson MedTech (USA), it is widely recognized for its versatility, ease of application, and ability to conform to irregular wound surfaces^22,23^. Composed of porcine-derived gelatin with a small-stellate appearance, SURGIFLO hemostatic matrix requires mixing with sterile saline prior to use. Currently, SURGIFLO hemostatic matrix holds a pivotal position in the market and is a widely used hemostatic product globally. On the other hand, Borayerflo hemostatic matrix is a Class III medical device developed by Jiangxi Borayer Biotechnology Ltd., which received approval from the National Medical Products Administration (NMPA) in the People’s Republic of China in 2023. It consists of a porcine-derived gelatin matrix with an expansion coefficient of less than 10%, minimizing the risk of nerve tissue compression while effectively controlling bleeding. Borayerflo’s unique flowable properties enable it to reach difficult-to-access oozing points, such as cavities, ensuring full contact with bleeding wounds. In this circumstance, the current study aims to compare the in vivo hemostatic efficacy of these two flowable gelatin-based hemostatic matrices using Beagle models with cerebral hemorrhage, an area that has been rarely explored in previous research. Therefore, this study addresses a significant gap in the literature by providing critical, head-to-head comparative data on hemostatic efficacy in a controlled Beagle model of cerebral hemorrhage, findings which could directly inform clinical practice and improve outcomes in neurosurgery.

## Materials and methods

### Materials

According to the manufacturer’s instructions, the SURGIFLO hemostatic matrix (8 mL, MS0010, Ethicon, Inc.) was prepared by mixing the gelatin matrix with 2 mL of sterile saline and then agitating the mixture through six reciprocal passes in two interconnected syringes. For the Borayerflo hemostatic matrix (8 mL, Jiangxi Borayer Biotech. Ltd.), the preparation involved mixing the gelatin powder with 7.2 mL of sterile saline, followed by 20 reciprocal passes in two interconnected syringes. Although Borayerflo required 20 reciprocal passes for preparation compared to 6 for SURGIFLO, both were prepared in less than 2 min and typically before surgery. Subsequently, the hemostatic matrix was applied to the bleeding site until the entire wound was fully covered, and a gauze dressing was placed over it to ensure complete contact between the hemostatic agent and the bleeding tissue. After 2 min, the gauze was removed, and the bleeding site was examined for hemostasis. Upon achieving hemostasis, any excess hemostatic gelatin was gently rinsed away with saline to prevent disruption of the clot. In the blank control group, bleeding was controlled solely by using cotton gauze (Zhende Medical Co., Ltd) pressure.

### Animals

Twenty-eight Beagles (14 females, 14 males) were randomly assigned to three experimental groups: SURGIFLO (*n* = 12), Borayerflo (*n* = 12), and gauze control (*n* = 4). Each group was sacrificed at 2, 4, 6, and 8 weeks post-implantation, with 3 dogs per group per time point for SURGIFLO and Borayerflo to ensure robust histological analysis. The gauze group (*n* = 4) served as a baseline control at each time point to verify tissue response without active hemostatic agents. The dogs weighed between 9 and 12 kg and were 9–15 months old. Prior to the commencement of any procedures, all animals underwent a minimum 7-day acclimation period to the testing environment. Throughout the study, the dogs were fed an appropriate quantity of dog food twice daily and had unrestricted access to drinking water. If any intraoperative, unmanageable complications arose, the affected animal would be euthanized (intravenous injection of 10% potassium chloride (KCl) at a dose of 0.5 ml/kg) under anesthesia (intravenous injection of Zoletil at a dose of 5 mg/kg and followed by the inhalation of 1–5% isoflurane)^24^. The Institutional Animal Care and Use Committee (IACUC) application has been rigorously reviewed by the Animal Center Laboratory Animal Management and Use Committee (Shanghai Xinova Medical Research Co., Ltd) prior to the initiation of the experiment, and animal testing will commence only after approval. The study protocol was approved by the Institutional Review Board of Shanghai Xinova Medical Research Co., Ltd (Reference #: XNYX23-122). During the experiment, veterinarians are responsible for monitoring the overall welfare of the animals. All methods were performed in accordance with the relevant guidelines and regulations. The study is reported in accordance with ARRIVE guidelines.

### Cerebral hemorrhage models

Anesthesia was induced with an intravenous administration of Zoletil at a dose of 5 mg/kg. Following successful tracheal intubation, the animals were promptly transferred to the operating table and connected to an anesthesia machine. Anesthesia was maintained through the inhalation of 1–5% isoflurane. The anesthetized dog was placed in a prone position, and an incision was made along the sagittal suture on the top of the Beagle’s head to dissect through the skin and subcutaneous tissues. Subsequently, the muscles overlying the skull were carefully retracted to expose the cranium. A bone window was created starting from the junction of the parietal and sagittal crests, extending 1.5–2.5 cm anteriorly towards the face, and then expending 0.5–1.5 cm laterally to the right. Herein, to eliminate confounding bleeding from skin and bone, we employed a staged hemostasis protocol. We ensured that the hemostasis of skin and bone was achieved prior to dural incision. Then, the dura mater was carefully incised to expose the parietal lobe of the brain. The Target Bleeding Site (TBS) was defined as a standardized area on the cortical surface located approximately 5 mm lateral to the sagittal suture and 10 mm anterior to the lambdoid suture. This specific location was selected to avoid major superficial vasculature and critical functional areas. Hemostasis was meticulously achieved prior to clamping the cerebral cortex, ensuring that the clamped area did not exceed 3 × 3 mm in size, with a clamping depth no greater than 2 mm. This procedure is technically straightforward, has no adverse effects, and demonstrates a high success rate.

### Hemostasis time

**Fig. 1:**
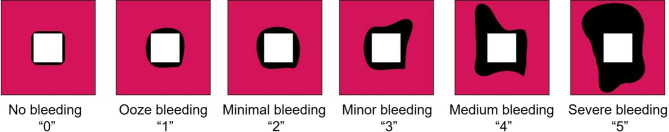
A graded scale was employed to evaluate the extent of bleeding from each lesion, with “no bleeding” and “ooze bleeding” categorized as successful hemostasis.

After successfully establishing the brain hemorrhage models in Beagle dogs, the SURGIFLO and Borayerflo hemostatic matrices (~ 1 mL) were administered to the bleeding site via a catheter, in accordance with the manufacturer’s instructions. After the application of hemostatic matrices, the site was then covered with gauze dressing and pressed to ensure complete contact of hemostatic matrices with the bleeding wound. The gauze was removed at predetermined intervals to evaluate if the hemostasis had been achieved. Successful hemostasis was defined as ‘no bleeding’ (Grade 0: no visible blood for ≥ 1 min after saline challenge) or ‘ooze bleeding’ (Grade 1: non-pulsatile droplets forming at ≤ 1 droplet/10 sec without pooling). Grades 0–1 were aggregated as success based on surgical standards^17^ (Fig. 1). The hemostatic effect was evaluated, and the time to achieve hemostasis was recorded.

### Blood tests

Given the critical role of blood tests in assessing the efficacy of hemostatic agents, comprehensive physical examinations and serum biochemical analyses were conducted on each dog both preoperatively and post-euthanasia. Furthermore, specific coagulation parameters including fibrinogen (FIB) levels, thrombin time (TT), prothrombin time (PT), International Normalized Ratio (INR), and activated partial thromboplastin time (APTT) were quantified.

### Toxicity evaluation

To assess the potential toxicity of SURGIFLO and Borayerflo hemostatic matrices, Beagles undergoing hemostasis with these agents were euthanized at 2, 4, 6, and 8 weeks post-implantation. Euthanasia was performed via intravenous injection of 10% KCl (0.5 ml/kg) following anesthesia with intravenous Zoletil (5 mg/kg) and 1–5% isoflurane inhalation. Heart, liver, spleen, lung, kidney, and brain tissues were collected and analyzed using H&E staining. Additionally, Nissl staining was performed on the brain tissue at the surgical site to assess whether either hemostatic agent caused neuronal damage.

### Statistical analysis

The log-rank test was used to compare the survival distributions related to hemostasis between the SURGIFLO and Borayerflo groups. The Wilcoxon rank-sum test was applied to assess changes in coagulation parameters before and after surgery within each group. A statistically significant difference was indicated by *p < 0.05, **p < 0.01, and ***p < 0.001.

## Results and discussion

### Hemostatic efficacy

**Fig. 2:**
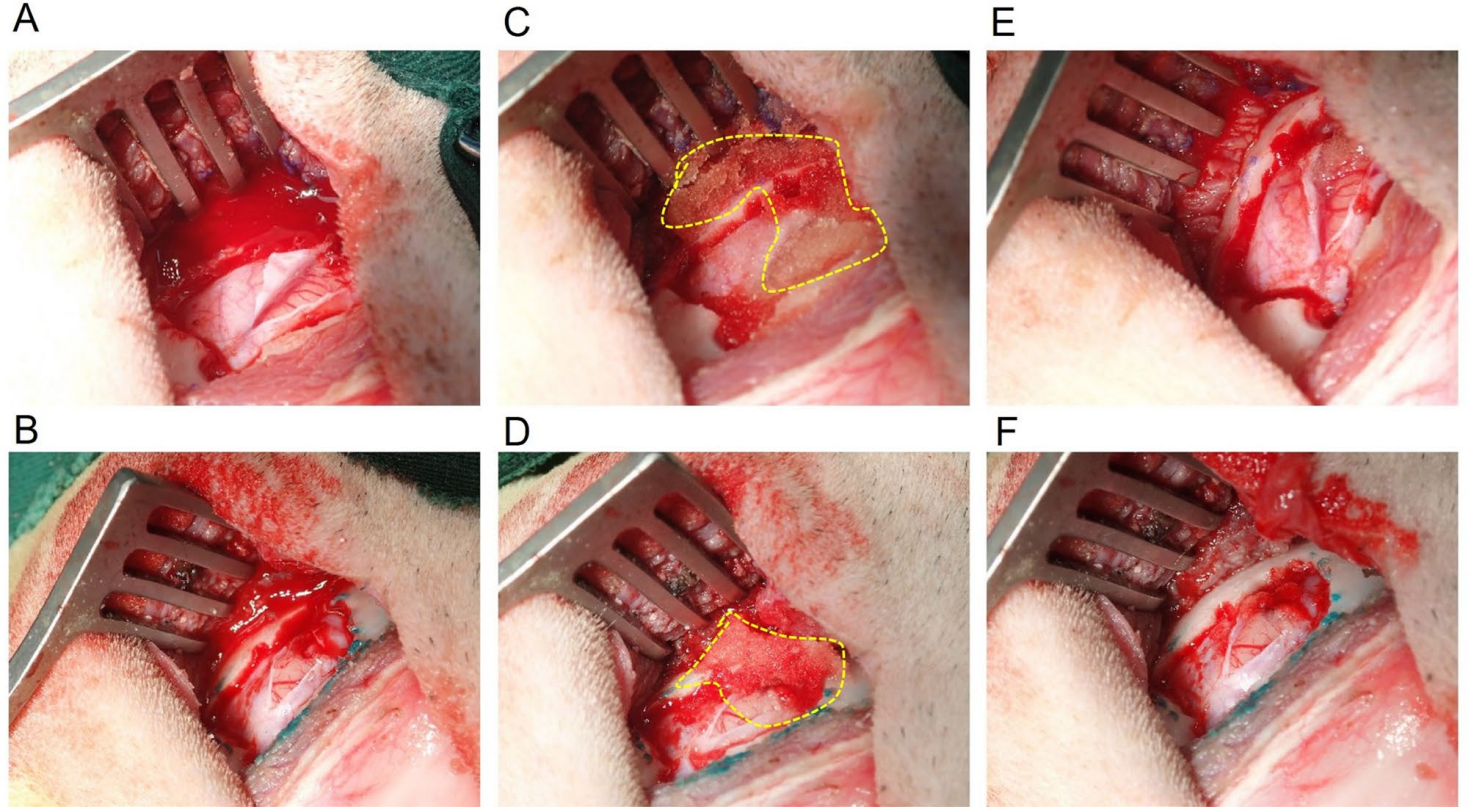
Application of flowable gelatin-based hemostatic matrices in a 3 × 3 cm, 1.5 cm deep cerebral hemorrhage model in Beagles. (**A**, **B**) Burr hole sites before treatment for (**A**) SURGIFLO and (**B**) Borayerflo groups. (**C**, **D**) Successful hemostasis after application of (**C**) SURGIFLO and (**D**) Borayerflo matrices, with matrices (slightly yellow) outlined by yellow dotted lines for clarity due to tissue integration. (**E**, **F**) Post-hemostasis images following saline rinse to remove excess material for (**E**) SURGIFLO and (**F**) Borayerflo groups.

**Fig. 3:**
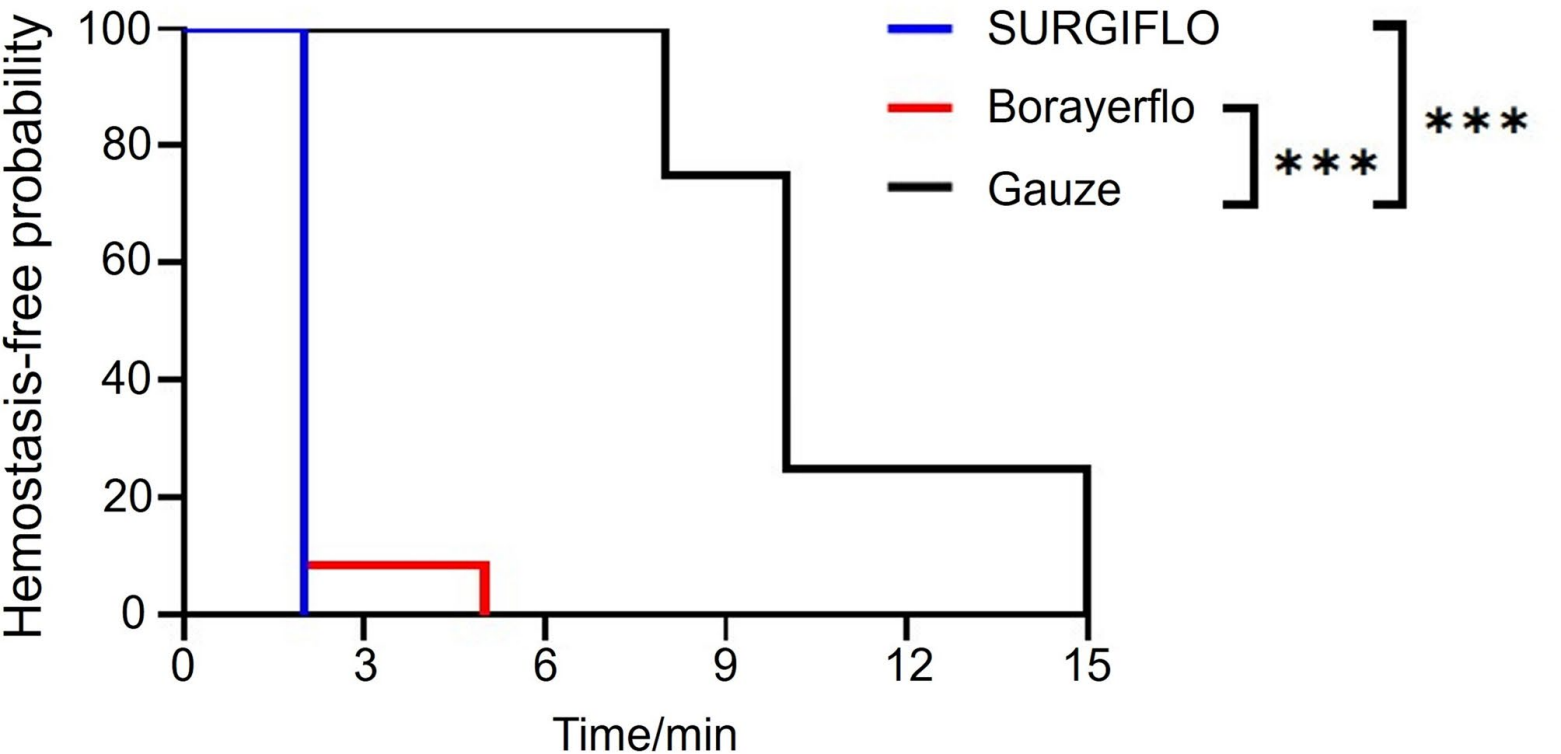
Product-Limit Survival Estimates (Kaplan–Meier [KM] curves): A log-rank test was conducted to compare the survival distributions of hemostasis between the SURGIFLO and Borayerflo groups. A decrease in the KM curve indicating a lower survival probability, suggests effective hemostasis, whereas a higher survival probability indicates ongoing bleeding (SURGIFLO group, *n* = 12; Borayerflo groups, *n* = 12; gauze group, *n* = 4).

As shown in Figs. 2 and 3, all bleeding sites treated with SURGIFLO matrix (12/12, 100%) achieved hemostasis within 2 min, demonstrating the high efficacy of SURGIFLO matrix for hemostasis, which is consistent with previous studies^23,25^. For example, Hutchinson et al. previously demonstrated that both Surgiflo and Floseal matrices could achieve hemostasis within 3 min at all sites regardless of the level of initial bleeding^25^. Additionally, Landi et al. indicated that complete hemostasis was achieved in all patients within 5 min in bleeding control during thoracic and lumbar spine surgery while using the Surgiflo matrix in a clinical trial^9^. A comparative study also revealed that the absolute time to hemostasis of the Surgiflo matrix was within 5 min in a porcine spleen biopsy punch model of moderate bleeding^24^. In contrast, 11 out of the 12 sites (91.67%) treated with the Borayerflo matrix achieved hemostasis within 2 min. Complete hemostasis was reached within 5 min (Fig. 3). The Kaplan-Meier curves in Fig. 3 further demonstrated that the hemostatic effects of Borayerflo and SURGIFLO were comparable in cerebral hemorrhage models in Beagle dogs, with no statistically significant differences observed between the two. Furthermore, it was evident that the hemostatic efficacy of both Borayerflo and SURGIFLO agents was significantly higher than that of the gauze group.

### Blood tests

To further evaluate the hemostatic efficacy of SURGIFLO and Borayerflo agents, blood tests were conducted. Fibrinogen, a soluble plasma glycoprotein, is an indispensable component of hemostasis, playing a critical role in clot formation and stabilization. As the precursor of fibrin, fibrinogen forms the structural framework of a blood clot. Generally, thrombin is activated through a cascade of enzymatic reactions triggered by tissue injury or exposure to a foreign surface^26^. As shown in Table 1, both matrices preserved FIB levels within normal ranges pre- and post-surgery, indicating minimal consumption of fibrinogen and efficient clot reinforcement. Moreover, there were no significant differences in fibrinogen levels between the Borayerflo (3.133 ± 0.675 vs. 2.151 ± 0.507) and SURGIFLO (2.739 ± 0.668 vs. 2.443 ± 1.311) groups both pre- and post-surgery, suggesting the comparable functions of these two hemostatic matrices.

**Table 1:** Changes in fibrinogen (FIB) level before and after surgery-quantitative analysis.

FIB (g/L)	Borayerflo	SURGIFLO	Total
Baseline			
N (Missing)	12 (0)	12 (0)	24 (0)
Mean (SD)	3.133 (0.675)	2.739 (0.668)	2.936 (0.687)
Median	3.085	2.550	2.835
Q1, Q3	2.755, 3.543	2.293, 3.163	2.385, 3.278
Min, Max	2.140, 4.630	2.050, 4.390	2.050, 4.630
P-value	0.104		
Postoperative			
N (Missing)	12 (0)	12 (0)	24 (0)
Mean (SD)	2.151 (0.507)	2.443 (1.311)	2.297 (0.984)
Median	1.990	2.065	2.040
Q1, Q3	1.723, 2.580	1.423, 3.085	1.710, 2.655
Min, Max	1.600, 3.240	1.150, 5.790	1.150, 5.790
P-value	0.943		

N: the number of Beagle dogs; SD: standard deviation; Q1: the lower quartile; Q3: the upper quartile; Min: the minimum; Max: the maximum.

Thrombin time (TT) is a straightforward coagulation assay that quantifies the conversion of fibrinogen to fibrin upon the addition of a thrombin reagent. TT assesses the efficiency with which thrombin in the hemostatic matrix converts fibrinogen to fibrin, thereby providing insights into the hemostatic matrix’s functional integrity. Therefore, TT is frequently employed to screen for both qualitative and quantitative fibrinogen deficiencies, evaluate fibrin formation disorders, and assist in the diagnosis of patients with prolonged prothrombin time (PT) and activated partial thromboplastin time (APTT)^27^. A prolonged TT can indicate issues such as fibrinogen depletion or the presence of inhibitors that may impair the hemostatic matrix’s functionality. Table 2 shows that there were no significant differences in TT between the Borayerflo (9.678 ± 0.869 vs. 10.670 ± 1.548) and SURGIFLO (9.263 ± 0.792 vs. 14.190 ± 14.350) groups, both pre- and post-surgery, indicating comparable efficacy of the two hemostatic matrices.

**Table 2:** Changes in thrombin time (TT) before and after surgery-quantitative analysis.

TT/s	Borayerflo	SURGIFLO	Total
Baseline			
N (Missing)	12 (0)	12 (0)	24 (0)
Mean (SD)	9.678 (0.869)	9.263 (0.792)	9.470 (0.840)
Median	10.030	8.790	8.955
Q1, Q3	8.840, 10.150	8.720, 10.020	8.723, 10.150
Min, Max	8.600, 11.410	8.600, 10.890	8.600, 11.410
P-value	0.213		
Postoperative			
N (Missing)	12 (0)	12 (0)	24 (0)
Mean (SD)	10.670 (1.548)	14.190 (14.350)	12.430 (10.140)
Median	10.530	10.190	10.240
Q1, Q3	9.218, 11.620	8.750, 11.680	8.975, 11.680
Min, Max	8.700, 14.110	8.600, 59.600	8.600, 59.600
P-value	0.724		

N: the number of Beagle dogs; SD: standard deviation; Q1: the lower quartile; Q3: the upper quartile; Min: the minimum; Max: the maximum.

The Prothrombin Time (PT) assay is widely used in hemostasis laboratories to measure the time required for clot formation following the addition of tissue factor (thromboplastin) and calcium to plasma^28^. Given that many hemostatic matrices either contain or promote the release of tissue factor, PT can effectively evaluate how these matrices enhance the extrinsic coagulation pathway to initiate clot formation. A shorter PT in the presence of a hemostatic matrix indicates its effectiveness in accelerating the coagulation process. The International Normalized Ratio (INR, derived from PT, serves as a standardized method to assess the efficacy of a hemostatic matrix. Consequently, PT and INR are routinely employed to evaluate a patient’s coagulation status and guide clinical treatment decisions^29^. The blood test results in Tables 3 and 4 also revealed that no significant differences in PT and INR were observed between the two groups before and after surgery.

**Table 3:** Changes in prothrombin time (PT) before and after surgery-quantitative analysis.

PT/s	Borayerflo	SURGIFLO	Total
Baseline			
N (Missing)	12 (0)	12 (0)	24 (0)
Mean (SD)	8.144 (0.555)	8.016 (0.429)	8.080 (0.489)
Median	7.985	7.950	7.985
Q1, Q3	7.843, 8.515	7.815, 8.145	7.823, 8.148
Min, Max	7.270, 9.320	7.210, 8.820	7.210, 9.320
P-value	0.579		
Postoperative			
N (Missing)	12 (0)	12 (0)	24 (0)
Mean (SD)	8.093 (0.360)	8.090 (0.480)	8.091 (0.414)
Median	7.940	7.945	7.940
Q1, Q3	7.893, 8.055	7.813, 8.633	7.890, 8.070
Min, Max	7.890, 9.010	7.410, 8.830	7.410, 9.010
P-value	0.831		

N: the number of Beagle dogs; SD: standard deviation; Q1: the lower quartile; Q3: the upper quartile; Min: the minimum; Max: the maximum.

**Table 4:** Changes in international normalized ratio (INR) before and after surgery-quantitative analysis.

INR	Borayerflo	SURGIFLO	Total
Baseline			
N (Missing)	12 (0)	12 (0)	24 (0)
Mean (SD)	0.692 (0.045)	0.682 (0.035)	0.687 (0.040)
Median	0.680	0.675	0.680
Q1, Q3	0.670, 0.720	0.663, 0.690	0.670, 0.690
Min, Max	0.620, 0.790	0.620, 0.750	0.620, 0.790
P-value	0.595		
Postoperative			
N (Missing)	12 (0)	12 (0)	24 (0)
Mean (SD)	0.687 (0.029)	0.689 (0.040)	0.688 (0.034)
Median	0.675	0.675	0.675
Q1, Q3	0.670, 0.688	0.670, 0.735	0.670, 0.690
Min, Max	0.670, 0.760	0.630, 0.760	0.630, 0.760
P-value	0.993		

N: the number of Beagle dogs; SD: standard deviation; Q1: the lower quartile; Q3: the upper quartile; Min: the minimum; Max: the maximum.

Thrombin generation is a critical process in the formation of a hemostatic plug or thrombus^30^. The coagulation system is typically evaluated using two standard in vitro clotting tests: activated partial thromboplastin time (APTT) and PT. APTT and PT assess the interconnection of components in plasma coagulation, delineating the intrinsic and extrinsic pathways, which converge at the formation of the prothrombinase complex. APTT is an important blood coagulation test used to assess the intrinsic and common pathways of the blood clotting cascade. It quantifies the time, in seconds, required for blood to clot after the addition of specific reagents to a plasma sample. The normal APTT range is typically between 30 and 40 s, although this can vary depending on the laboratory’s standards and methodologies. As shown in Table 5, preoperative APTT times were similar in the Borayerflo (7.49 ± 2.65) and SURGIFLO (6.95 ± 2.07) groups. Postoperatively, there was a minor decrease in APTT (5.19 ± 1.05) in the SURGIFLO (5.29 ± 2.28) group; however, such decreases are uncommon and often regarded as spurious^31^. Therefore, the observed difference in postoperative APTT levels warrants further validation in the follow-up studies. Likely, the post-operative APTT decrease may reflect preanalytic artifacts (e.g., citrate effects) or true hypercoagulability from surgical stress. Moreover, previous study has reported a shortened APTT in hypercoagulable states^32^.

**Table 5:** Changes in activated partial thromboplastin time (APTT) before and after surgery-quantitative analysis.

APTT/s	Borayerflo	SURGIFLO	Total
Baseline			
N (Missing)	12 (0)	12 (0)	24 (0)
Mean (SD)	7.490 (2.650)	6.950 (2.070)	7.220 (2.440)
Median	6.640	6.330	6.500
Q1, Q3	5.940, 7.580	5.590, 7.120	5.820, 7.380
Min, Max	4.740, 15.080	5.100, 12.970	4.740, 15.080
P-value	0.600		
Postoperative			
N (Missing)	12 (0)	12 (0)	24 (0)
Mean (SD)	5.190 (1.050)	5.290 (2.280)	5.240 (1.820)
Median	5.360	4.800	5.180
Q1, Q3	5.010, 5.480	3.360, 6.610	4.470, 5.620
Min, Max	2.440, 7.230	2.540, 9.760	2.440, 9.760
P-value	0.900		

N: the number of Beagle dogs; SD: standard deviation; Q1: the lower quartile; Q3: the upper quartile; Min: the minimum; Max: the maximum.

### Toxicity study

**Fig. 4:**
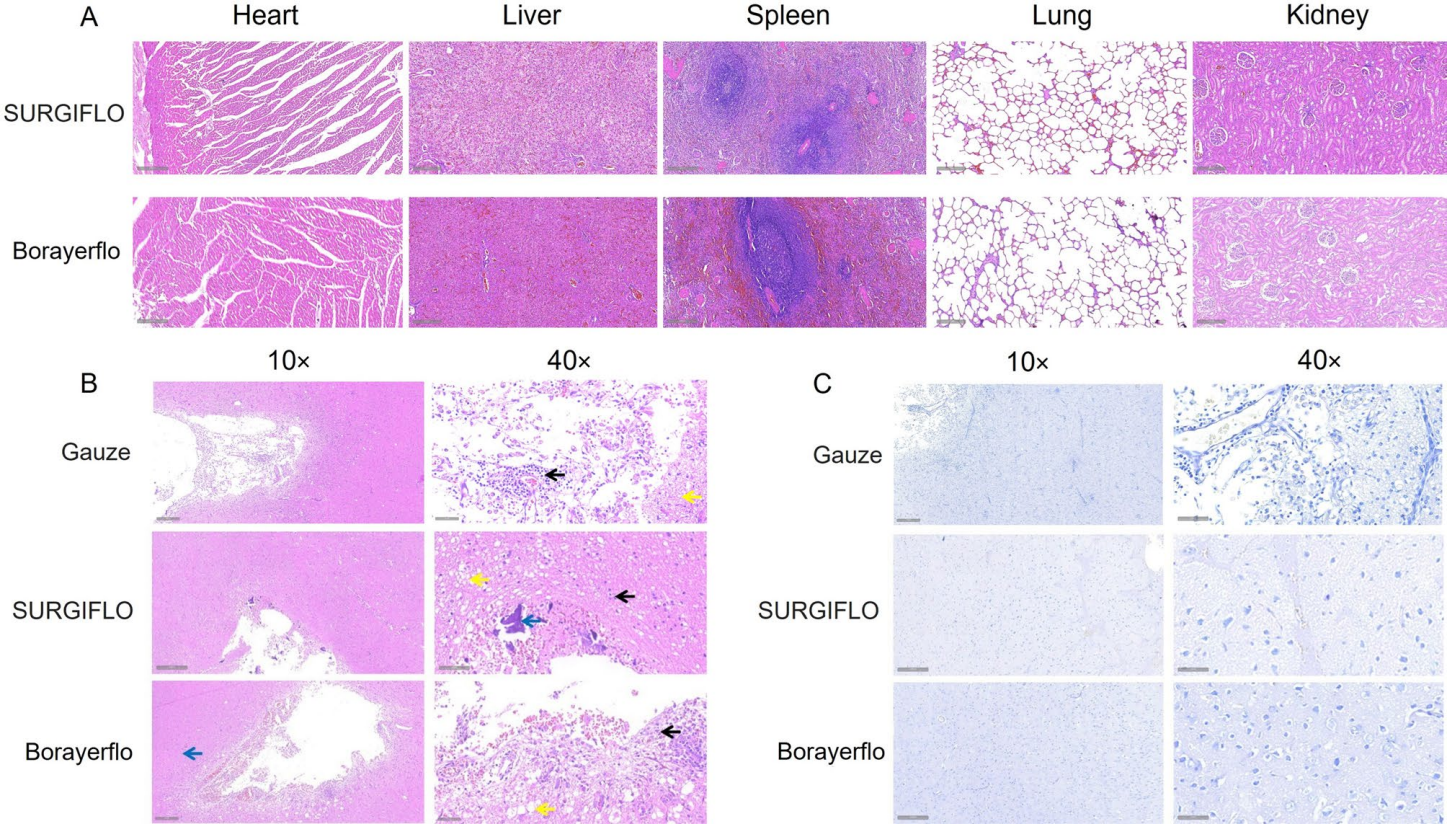
Histological results of major organs of Beagle dogs at 4 weeks post-operation. (**A**) H&E staining images of major organs of Beagle dogs after hemostasis (10×, scale bar: 200 μm). (**B**) H&E staining images of surgical brain sites using SURGIFLO and Borayerflo matrices for hemostasis (10×, scale bar: 200 μm; 40×, scale bar: 50 μm). (**C**) Nissl staining images of surgical brain sites using SURGIFLO and Borayerflo matrices for hemostasis (10×, scale bar: 200 μm; 40×, scale bar: 50 μm).

The vital organs (heart, liver, spleen, lung, and kidney) of Beagle dogs were sectioned and stained (Fig. 4A). The H&E staining results revealed no significant changes in the major organs of Beagle dogs in both the SURGIFLO and Borayerflo groups, indicating that these hemostatic matrices do not induce significant toxicity or adverse effects on the organs. Considering the well-established biocompatibility of gelatin and its widespread use in tissue engineering applications, it is reasonable to infer that the SURGIFLO and Borayerflo hemostatic matrices are biocompatible for clinical applications.

Histological analysis of brain tissue at the surgical site was conducted at 2, 4, 6, and 8 weeks post-implantation using H&E staining, evaluated per ISO 10993-6 standards: [(polymorphonuclear cells + lymphocytes + plasma cells + macrophages + giant cells + necrosis) × 2 + neovascularization + fibrosis + fatty infiltration]. Irritation grades are: none (0.0–2.9), slight (3.0–8.9), moderate (9.0–15.0), severe (≥ 15.1). At 2 weeks, Borayerflo and SURGIFLO groups showed focal hemorrhage, inflammatory cell infiltration, interstitial edema, occasional neovascularization, and reduced Nissl body counts (neuronal damage), with irritation scores of 5.3 (Borayerflo) and 5.7 (SURGIFLO; slight irritation). The blank control group had vascular congestion, reduced neuron counts, and a higher score (7.0). At 4 weeks, no hemorrhage was observed, with minimal residual implant material (Fig. 4B, blue arrows), consistent with near-complete resorption by 6 weeks (confirmed in rabbit models). SURGIFLO showed minimal inflammatory cell infiltration (black arrows), interstitial edema (yellow arrowheads), reduced neuron/Nissl body counts, and rare fibrous tissue proliferation (score: 6.7; slight irritation). Borayerflo exhibited moderate inflammatory cell infiltration (score: 3.0; black arrowheads), with similar findings (score: 6.7). The blank control group showed neuronal necrosis, reduced Nissl bodies, and moderate irritation (score: 9.0). At 6 weeks, both groups had minimal edema, inflammation, and thin fibrous layers, with scores of 2.6 (Borayerflo) and 4.0 (SURGIFLO). The blank control group showed mild hemorrhage, edema, and inflammation (score: 8.0). By 8 weeks, both groups exhibited minimal inflammation, edema, and reduced Nissl bodies, with no implant material (Borayerflo: 3.0; SURGIFLO: 1.3; blank control: 5.0). These findings reflect normal healing, with Borayerflo and SURGIFLO reducing inflammation and necrosis compared to the blank control. Neither agent caused additional brain tissue damage, and tissue repair was evident, consistent with their 4–6-week resorption.

Moreover, Guo et al. have indicated that SURGIFLO could serve as neuroprotective agents for reducing inflammatory reactions in secondary brain injury and is suitable for the clinical use in traumatic brain injury (TBI) patients^33^. The Nissl staining method is normally used to visualize neurons and assess cellular synthetic activity by highlighting chromatophilic substances, including ribosomal RNA in the rough endoplasmic reticulum and polyribosomes^34,35^. As shown in Fig. 4C, in the SURGIFLO group, a mild reduction in the number of Nissl bodies was observed both at and around the surgical site. In contrast, the Borayerflo group showed a higher abundance of Nissl bodies in and around the surgical area. Overall, Borayerflo and SURGIFLO hemostatic matrices were biocompatible for clinical use.

### Limitations

This study has several limitations. First, the sample size of the gauze control group (*n* = 4) was small and precludes a powerful statistical comparison to the treatment groups. Its primary role was to serve as a procedural control, confirming the validity of the hemorrhage model. Future studies with larger control groups could provide more robust statistical benchmarks for efficacy. Second, the histological analysis was conducted with a small subgroup size (*n* = 3 per group per time point) to track temporal responses. While this allowed for the observation of clear trends (such as the more pronounced inflammation in the Borayerflo group), this sample size provides low statistical power. The histological findings should therefore be interpreted as descriptive and indicative of the healing trajectory rather than as definitive quantitative outcomes. Future studies focusing on a specific endpoint should utilize larger group sizes to enable robust statistical comparison of tissue response. Last, the hemostatic efficacy was evaluated in a healthy animal model. This does not replicate the clinical reality of many neurosurgical or trauma patients who may present with underlying coagulopathies (e.g., due to anticoagulant medication, liver dysfunction, or massive transfusion). The performance of the hemostatic agents might differ under such impaired hemostatic conditions, and future studies in anticoagulated models would be valuable to assess efficacy in a more challenging physiological context.

## Conclusions

The current study demonstrated that the Borayerflo hemostatic matrix exhibited comparable hemostatic efficacy to the SURGIFLO hemostatic matrix in terms of hemostasis efficacy and safety in a cerebral hemorrhage model using Beagle dogs. Specifically, the absolute time to hemostasis and blood test results, including FIB levels, TT, PT, INR, and APTT, showed no statistically differences between Borayerflo and SURGIFLO hemostatic matrices, supporting the potential equivalence in clinical performance. Moreover, both Borayerflo and SURGIFLO hemostatic matrices did not induce toxicity or adverse effects on the vital organs. Consequently, the Borayerflo hemostatic matrix holds significant potential as an absorbable topical hemostatic agent in the market.

## Data Availability

Data will be made available on request. Requests should be directed to the Corresponding Author at shangsichen@cuhk.edu.hk.
